# Is Cardiopulmonary Fitness Related to Attention, Concentration, and Academic Performance in Different Subjects in Schoolchildren?

**DOI:** 10.3390/jfmk10030272

**Published:** 2025-07-16

**Authors:** Markel Rico-González, Ricardo Martín-Moya, Jorge Carlos-Vivas, Francisco Javier Giles-Girela, Luca Paolo Ardigò, Francisco Tomás González-Fernández

**Affiliations:** 1 Department of Didactics of Musical, Plastic and Corporal Expression, University of the Basque Country—UPV-EHU, 48940 Leioa, Spain; markel.rico@ehu.eus; 2 Department of Physical Education and Sports, Faculty of Education and Sport Sciences, Campus of Melilla, University of Granada, 52006 Melilla, Spain; rmartinm@ugr.es (R.M.-M.); javiggr@hotmail.com (F.J.G.-G.); ftgonzalez@ugr.es (F.T.G.-F.); 3 Physical Activity for Education, Performance and Health (PAEPH) Research Group, Faculty of Sport Sciences, University of Extremadura, 10003 Cáceres, Spain; jorgecv@unex.es; 4 Department of Teacher Education, NLA University College, 0166 Oslo, Norway

**Keywords:** sport, exercise, correlation, cognition, children

## Abstract

**Background:** The perceived importance of physical practice and its contribution to students’ academic success have evolved considerably throughout the history of the modern educational system. **Aim:** The purpose of this study was to understand the relationship between physical fitness (measured as VO_2_max) and cognitive abilities (attention and concentration) and academic performance in different subjects: sciences, letters, language, arts, and physical education. **Method:** Fifty Spanish male students who participated in extracurricular sports activities (mean age (SD): 11.59 ± 1.30; range: 9–15 years) were included in the analysis. The 6 min walk test was used to assess physical fitness (6MWT), while for selective attention and concentration, the students completed the D2 test, which is usually considered to analyse the visual ability to select the most relevant stimulus of an exercise and ignore precisely the most irrelevant stimuli. **Results:** Correlation the individual contribution analyses revealed no significant associations between VO_2_max and academic performance in sciences (r = 0.04, *p* = 0.77), humanities (r = 0.00, *p* = 0.98), language (r = 0.03, *p* = 0.83), or arts (r = 0.04, *p* = 0.76). Similarly, no relationship was found between VO_2_max and overall academic performance (r = 0.10, *p* = 0.46), or cognitive abilities. However, a small positive correlation was observed between VO_2_max and physical education scores. **Conclusions:** Physical fitness showed no significant association with cognitive abilities or academic performance in most subjects, although a small positive correlation with physical education scores was observed. These findings emphasise the importance of promoting physical activity for its health and physical benefits. However, future research should explore broader cognitive outcomes and include more diverse and representative samples.

## 1. Introduction

The health of school-aged children is influenced by biological, behavioural, and environmental factors [1]. Most research related to this field focuses on the effects of healthy eating, physical activity (PA), sedentary behaviour, and sleep quality [2,3,4]. Recent studies have shown that only a few children meet recommendations for daily PA [5,6]. In addition, sedentary behaviour is increasing in children and adolescents due to the overuse of new technologies [7].

The effect of PA on the health of schoolchildren is widely recognised [8,9]. Regular exercise improves anthropometric measures such as weight and body composition [10]. In addition, regular exercise has a positive effect on numerous health indicators in children and adolescents, including physical condition, cardiometabolic and bone health [11,12], cognitive abilities (e.g., academic performance), and mental health [13,14,15]. Physical exercise has been shown to trigger changes in the human brain owing to increased metabolism, oxygenation, and blood flow, which generate hormones that promote neurological health [16]. Children’s health is also influenced by behavioural and cognitive factors [17].

Contemporary educational organisations propose that children’s experiences in sport and physical education (PE) contribute to the development of skills and strategies that are important to address the challenges they face throughout life [18]. In this context, educational failure has also become a major problem for young people of school age, so it is very important to promote healthy lifestyle habits that indirectly improve academic performance [19]. Specifically, the imprint of physical exercise has been shown on certain factors that influence academic performance, such as memory, attention span, and executive functions [17,20], due to the decrease that exercise produces in cortisol concentrations (a hormone related to attention deficit), the creation of endorphins to generate attitudes more conducive to learning [21], and better blood supply to the brain, which strengthens the stimulation of neurotrophic factors [22]. Therefore, recommendations for exercise are generated, for example, by the World Health Organisation for this population group [23].

The perceived importance of physical practice and its contribution to students’ academic success have evolved considerably throughout the history of the modern educational system [24]. Studies have reported positive associations between concentration (as a cognitive factor) and physical fitness in children aged 10–12 years [15,25]. In addition, greater physical capacity seems to be related to better academic performance [26,27]. In the long term, the development of this ability during childhood is positively associated with higher levels of concentration in adulthood [28]. In summary, associations have been shown between the practice of physical exercise, the ability to maintain concentration and quality of life in schoolchildren and adolescents [24,26].

In this sense, and following Tomporowski et al., they affirmed that the influence of physical exercise on academic performance can be extended through the impact of physical practice on aerobic fitness [29]. Aerobic fitness is an indicator of physical performance. Some studies have shown that aerobic fitness may not only promote cerebral blood flow to the hippocampus but also assist in the development of brain structure and function [30]. The authors agree that the practice of physical exercise and cardiorespiratory fitness is favourable for brain structure, brain cognition and function in children and young people [31]. They also showed that PA before, during, and after class can improve academic performance in children and adolescents, and that even a single session of moderate PA can have significant benefits on brain function, academic performance, and cognition [31]. In another study, eight leading researchers in the field of cognition [32] conducted a systematic review focusing on two specific questions: Do physical exercises affect brain function, learning, structure, and cognition among children aged 5–13 years? Do physical fitness and exercise programs affect performance and attention on standardised ability tests? They found promising outcomes showing a link between physical exercise, brain structure and function, and cognition, with no negative effects on schoolchildren.

It is still not clear how these measures (physical exercise, aerobic capacity, and cognitive capacity) interact and, consequently, how they influence attentional and concentration processes [20]. In this context, there is limited evidence on how behaviour change techniques moderate academic outcomes, highlighting the need for further investigation. Overall, it remains unclear whether physical activity interventions consistently impact academic performance through specific mediators at varying levels. Therefore, there is a need to investigate the association between aerobic fitness (VO_2_max) and cognitive performance—specifically selective attention and concentration—as well as academic achievement across core subjects (sciences, language, arts, and physical education) in physically active male school-aged students, thereby contributing to recent literature by clarifying these relationships within a homogeneously active population, where traditional associations may be attenuated or absent. This study adds to the recent literature by challenging commonly reported positive associations between physical fitness and cognitive or academic performance, suggesting that among physically active male students, aerobic fitness (VO_2_max) shows only a weak correlation with PE scores and no significant links with cognitive abilities or broader academic achievement—highlighting the need to consider contextual, psychosocial, and demographic factors in future research.

## 2. Materials and Methods

### 2.1. Participants

This study involved fifty-nine male students ranging from the fifth grade of elementary school to the second grade of secondary school. Participants were recruited from two schools in the Andalucía region and were required to meet the following inclusion criteria: (i) normal vision with no history of neuropsychological conditions that could influence the outcomes; (ii) absence of health issues that might bias results or prevent participation in the study assessments; and (iii) engagement in extracurricular sports activities.

Regarding sample size determination, a minimum of forty-two participants was necessary to achieve an estimated statistical power of 85.2%. This estimate was obtained through an a priori power analysis for a t-test focused on correlation analysis. The analysis assumed an alpha level of 0.05, a target power of approximately 84%, and a medium to large effect size based on previous research findings. All calculations were conducted using G*Power software version 3.1.9.7 (University of Düsseldorf, Germany).

Students were recruited from one village of the province of Jaén with a population ranging from 5000 to 10,000 inhabitants according to the National Institute of Statistics from the Spanish Government (http://www.ine.es/; accessed on 12 February 2022). Every school management member was contacted to request their participation. In addition, written informed consent was obtained from the parents or guardians of each adolescent who wanted to participate. The study was conducted in accordance with the Declaration of Helsinki and approved by the ethics committee of the institute’s research at the University of Granada (2021/89).

### 2.2. Measures and Instruments

#### 2.2.1. Physical Fitness

The 6 min walk test (6MWT) is a sub-maximal exercise assessment designed to evaluate aerobic capacity and endurance. Developed by the American Thoracic Society, the test is conducted in a corridor at least 30 m in length. The route’s length is marked every 3 m, with turn points designated by cones, and the starting position indicated using brightly coloured tape. Participants are instructed to walk back and forth along the corridor in a straight line as rapidly as possible, self-paced, over a six-minute period. Throughout the test, participants receive auditory prompts every minute to monitor their pace. Heart rate measurements are obtained using telemetry (Heart Rate Transmitter Model T34; Polar, Kempele, Finland) during the assessment. The total distance covered within the six minutes is recorded as the primary outcome measure. To establish the reliability of the 6MWT, a subset of 80 boys was randomly selected to repeat the test after a one-week interval. The consistency of their performance across the two assessments was then evaluated to confirm the test’s reproducibility [33].

#### 2.2.2. D2 Test

Students’ individual selective attention was measured using the D2 test [34], which is usually considered to analyse the visual ability to select the most relevant stimulus of an exercise and ignore precisely the most irrelevant stimuli. The test–retest reliability in the original study was up to 0.90.

Items: The D2 test comprised 658 elements divided into 14 rows, each of which included 47 characters. The items can be “p” or “d” with one, two, three, or four dashes, arranged either individually or in pairs at the top and/or bottom of each letter. Thus, the “d’s” with two dashes, regardless of position, should be crossed.

Time: Students were encouraged to mark as many of those d’s as possible in 20 s per row.

Evaluation: The scores are expressed as TR (processed elements), TA (successes), O (omissions), C (commissions or errors), TOT [effectiveness in the task = TR − (O + C)], CON (concentration = TA − C), TR+ (last stimulus analysed in the row with the most attempted elements), TR− (last stimulus analysed in the row with the least attempted elements), and VAR [index of variation between the last stimulus analysed between different rows = (TR+) − (TR−)].

### 2.3. Procedures

Physical fitness was evaluated using the 6MWT during the first day of physical education classes. The assessments were scheduled in the morning hours, between 9:00 a.m. and 1:30 p.m., and participants first completed a 10 min warm-up that included activation exercises and joint mobility movements. During the test, students were asked to walk freely along a designated path, and their performance was quantified by recording the total distance covered within the six-minute timeframe.

On the following day, attention and concentration were assessed under similar conditions, with testing times matched to ensure consistency across sessions. These cognitive evaluations involved standardised tasks specifically designed to measure focus and attentional capacity. Prior to testing, a detailed explanation of the procedures was provided to all students, and any questions were addressed by a trained educational psychologist, ensuring that participants understood the tasks and felt comfortable.

Academic performance data were obtained from the advisors of both participating schools. This information consisted of official academic records, such as report cards and transcripts, which were collected only with the explicit authorisation of the students or their guardians, in accordance with privacy regulations. To maintain consistency, these records were gathered within a window of 24 to 72 h relative to the other assessments. The scoring of each subject was carried out by teachers, who applied criteria provided beforehand to ensure transparency and consistency. Teachers were fully informed of the study’s objectives and the evaluation procedures, which helped preserve objectivity in assigning scores and aligned with the established guidelines.

Finally, the D2 test, a standardised instrument used to assess visual attention and processing speed, was administered following the formal testing protocol. Clear instructions were given to students, with the supervising psychologist offering clarifications as needed to prevent misunderstandings. The test was conducted in group settings within regular classroom environments, adhering strictly to the recommended procedures to uphold the test’s validity and reliability.

### 2.4. Statistical Analysis

Descriptive statistics were calculated for each variable. Normal distribution and homogeneity tests (Kolmogorov–Smirnov and Levene’s, respectively) were conducted on all metrics. The data were normally distributed. Subsequently, Pearson’s correlation coefficient *r* was used to examine the relationship between the VO_2_max and the final score of each subject group (total, sciences (SCI), letters (LETT), language (LAN), arts (ART), and PE), and D2 values (TR, TA, O, C, TR+, and TR−). To interpret the magnitude of these correlations, it was adopted the following criteria: r ≤ 0.1, trivial; 0.1 < r ≤ 0.3, small; 0.3 < r ≤ 0.5, moderate; 0.5 < r ≤ 0.7, large; 0.7 < r ≤ 0.9, very large; and r > 0.9, almost perfect. All analyses were conducted using Statistical software (version 13.1; Statsoft, Inc., Tulsa, OK, USA), and the significance level was set at *p* ≤ 0.05.

## 3. Results

Descriptive statistics for all measured variables, including anthropometric data, physical fitness, academic performance, and cognitive test scores, are presented in Table 1.

Regarding cognitive performance through the D2 Test of Attention, students demonstrated a mean total response TR score of 318.07, indicating their general attentional capacity. The average CON score was 111.31, suggesting a moderate level of sustained attention. However, the data also revealed considerable variability in other indices; for example, the O score (which reflects omission errors) averaged 27.00, and the C score (indicative of impulsivity or false alarms) averaged 9.32. These fluctuations point to differences in attentional control and inhibitory processes among participants, highlighting individual variability in cognitive regulation.

**Figure 1:**
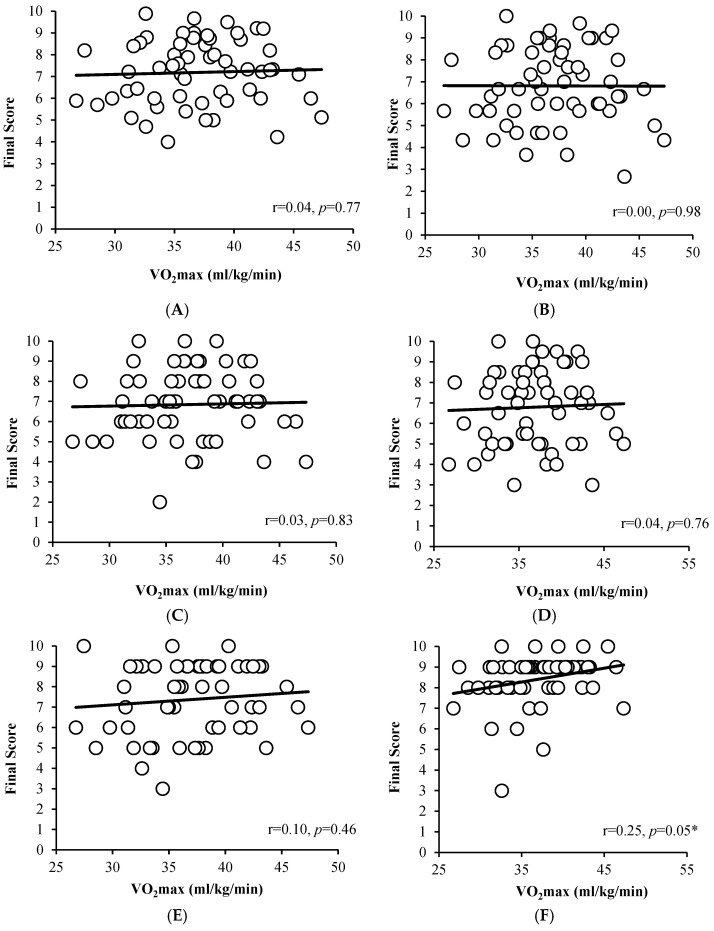
Correlation analysis between different subject values and VO_2_max (mL/kg/min). (**A**) Correlation between the final score (total) and VO_2_max (mL/kg/min). (**B**) Correlation between final score (SCI) and VO_2_max (mL/kg/min). (**C**) Correlation between the final score (LETT) and VO_2_max (mL/kg/min). (**D**) Correlation between the final score (LAN) and VO_2_max (mL/kg/min). (**E**) Correlation between final score (ART) and VO_2_max (mL/kg/min). (**F**) Correlation between the final score (PE) and VO_2_max (mL/kg/min). * significant correlation.

First, a correlation analysis was performed between VO_2_max and subject scores (total, SCI, LETT, LAN, ART, and the values did not reveal any correlation, r = 0.04, *p* = 0.77, r = 0.00, *p* = 0.98, r = 0.03, *p* = 0.83, r = 0.04, *p* = 0.76, r = 0.10, *p* = 0.46, respectively). However, we found a small positive correlation between VO_2_max and PE score (r = 0.25, *p* = 0.05). See Figure 1 for further details. Figure 1A–F visually illustrate these relationships, confirming the lack of meaningful trends between aerobic capacity and academic performance across most subjects, with the exception of PE.

**Figure 2:**
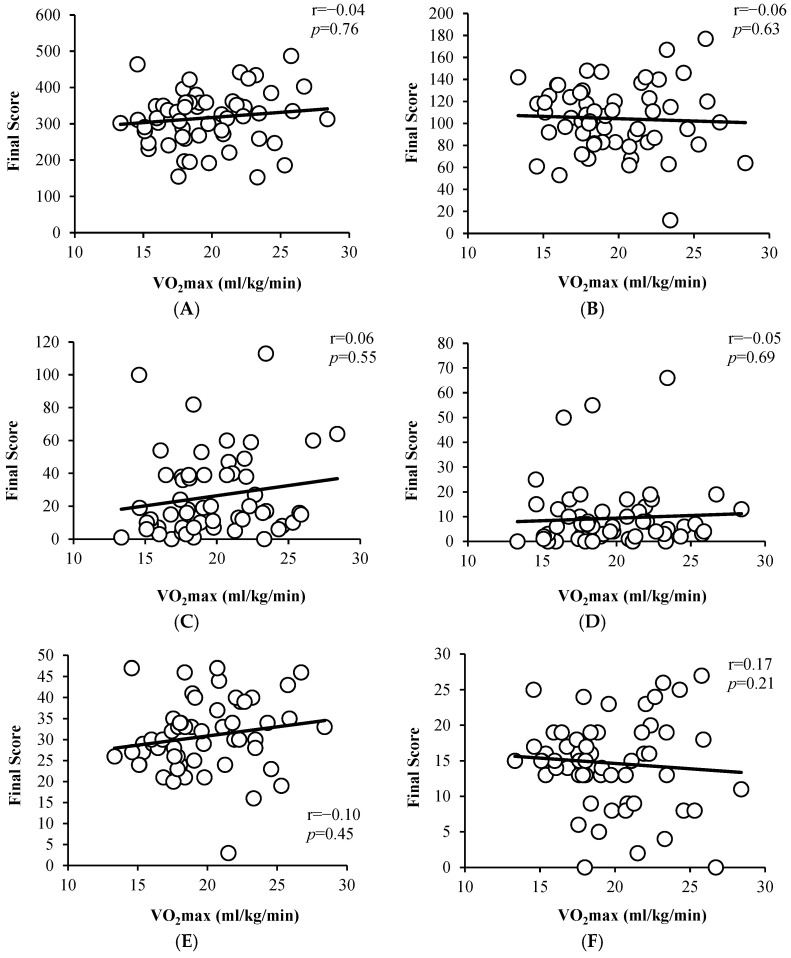
Correlation analysis between different D2 and VO_2_max values (mL/kg/min). (**A**) Correlation between TR and VO_2_max (mL/kg/min). (**B**) Correlation between TA and VO_2_max (mL/kg/min). (**C**) Correlation between O and VO_2_max (mL/kg/min). (**D**) Correlation between C and VO_2_max (mL/kg/min). (**E**) Correlation between TR+ and VO_2_max (mL/kg/min). (**F**) Correlation between TR− and VO_2_max (mL/kg/min).

A new correlation analysis was performed between VO_2_max and D2 test values (TR, TA, O, C, TR+ and TR−), crucially, the values did not reveal any correlation, r = −0.04, *p* = 0.76, r = −0.06, *p* = 0.63, r = 0.06, *p* = 0.66, r = −0.05, *p* = 0.69, r = −0.10, *p* = 0.45, and r = 0.17, *p* = 0.21, respectively. See Figure 2 for further details. These findings are detailed in Figure 2A–F and suggest no relationship between physical fitness and selective attention or concentration in this sample.

## 4. Discussion

The present study aimed to explore the association of physical fitness (measured as VO_2_max) with cognitive abilities (specifically, attention and concentration) and academic performance in sciences, letters, language, arts, and physical education.

In contrast to previous literature in the field [35,36,37], our findings revealed no relationships between physical fitness and cognitive abilities or between physical fitness and academic performance, except for PE score, where a small positive association was observed (r = 0.25, *p* = 0.05). These outcomes might be explained by the fact that our study only involved individuals who had to participate in extracurricular sports activities, while most of the previous studies in the topic included individuals with a wider and more heterogeneous spectrum regarding the daily behaviour related to physical activity. In fact, although previous studies informed that students who participate in regular physical activity and present higher levels of physical fitness tend to perform better academically than their less active and less fit peers [32,38]; recently, Honório et al., reported a moderate positive association between the academic performance and VO_2_max in students who practice physical exercise up to 3 h per week [39]; but not in those who practice physical exercise between 4 and 6 h per week, nor 7 or more hours of physical exercise per week. Thus, the fact that our participants regularly participated in extracurricular sports activities could have made finding clear associations more difficult.

Nevertheless, many studies have shown a positive correlation between physical fitness and academic achievement [27], suggesting that engaging in regular physical activity can improve cognitive abilities [15], which can lead to better academic performance. And the fact is that exercise increases blood flow to the brain, providing it with more oxygen and nutrients, promoting the growth of new neurons and the formation of new connections between them (i.e., neuroplasticity) [16,30]. This increased capacity for adaptation and learning can enhance cognitive abilities.

Specifically, the significant but weak correlation observed between PE score and VO_2_max may be due to the multifaceted nature of PE assessments. While VO_2_max reflects aerobic fitness, PE scores often encompass a broader range of skills, including motor coordination, technical ability, participation, and theoretical knowledge, which are not solely dependent on cardiorespiratory fitness. Furthermore, factors such as motivation, self-esteem, and engagement in class activities can influence PE performance independently of VO_2_max levels [1,2,3]. Therefore, the results should be interpreted with caution. The weak correlation suggests that interventions focused solely on increasing VO_2_max may have a limited impact on overall PE scores. Future research should consider the role of other physical fitness components, psychosocial factors, and the structure of PE curricula in shaping student outcomes. Moreover, the complexity of the relationship between fitness measures and performance underscores the necessity of comprehensive approaches in both research and educational practice [2,3,4]. In summary, while VO_2_max is an important indicator of aerobic fitness, its influence on PE scores is modest, reflecting the broader range of skills and attributes evaluated in physical education.

Physical activity can also help improve attention and focus, both of which are critical for academic success, making it easier to concentrate on tasks and process information more efficiently [17]. Exercise has been shown to increase the production of neurotransmitters such as dopamine and norepinephrine, which play essential roles in attention and focus [40]. Specifically, aerobic exercise increases the volume of brain regions involved in memory, learning, and executive functions. This can lead to improved cognitive abilities, including attention, memory, and problem-solving skills. Additionally, physical fitness can help reduce stress and anxiety, which can negatively impact attention and focus and subsequently affect academic performance [41].

Furthermore, physical activity can help improve sleep quality, which is essential for optimal cognitive function [41]. Lack of sleep quality can negatively affect cognitive abilities such as attention [42].

Likewise, regular physical activity can lead to improved self-esteem and motivation as it provides a sense of accomplishment which leads to increased feelings of self-worth and higher levels of confidence, thus enhancing overall well-being. Additionally, regular exercise promotes the release of endorphins, which increases feelings of happiness and improves mood state, leading to better motivation towards academic performance and resulting in improved grades [43,44]. Students who engage in regular physical activity may develop a positive self-image and feel more confident about their abilities. This increased self-esteem and motivation can, in turn, lead to better academic performance, as they increase confidence in one’s ability, resulting in higher levels of engagement in learning, which leads to improved grades. Students with high levels of motivation tend to be more persistent and focused on their goals, leading to enhanced problem-solving skills, thus enhancing overall academic success [45,46].

In summary, the current evidence seems to suggest that incorporating regular physical activity into a student’s routine is a crucial component of a healthy lifestyle and can contribute to improved cognitive abilities and overall academic performance. However, it is important to note that the relationship between physical fitness and academic achievement is not necessarily causal, and other factors such as age, gender, socioeconomic status, educational resources available in school or home environment, genetics, nutrition, etc., may also play a role. It is essential to recognise that everyone is different, and that the impact of physical activity on academic performance may vary from one individual to another. Moreover, the exact mechanisms by which physical activity and fitness influence academic performance are not fully understood, and further research is needed to completely understand the relationship between these two variables. Therefore, it is always best to consider multiple factors when evaluating an individual’s academic success, as there are many variables beyond physical fitness, such as emotional intelligence and social support systems, that can impact the overall performance of academics [47,48]. In this context, there is a need for more detailed research to understand how factors like age, gender, physical fitness, and body measurements affect cognitive function, particularly among specific groups such as Spanish students. For example, González-Fernández and colleagues [48] investigated these relationships with 187 students, emphasising the importance of taking into account various demographic and contextual factors. Ultimately, adopting a holistic approach that considers multiple influences will lead to a deeper understanding of the factors that contribute to academic success. Relying on a single variable, such as physical fitness or grades alone, is unlikely to capture the full picture, and a broader perspective is essential for developing effective strategies to improve educational outcomes.

Some limitations of the present study warrant careful consideration. Its cross-sectional design precludes any definitive conclusions regarding the directionality of the observed associations, thus limiting causal inference. The reliance on a convenience sampling approach further restricts the generalizability of our findings, as the sample may not accurately represent the broader population of students. Additionally, the sample comprised only male students from two schools, spanning from the fifth grade of elementary education to the second grade of secondary education, all of whom participated in at least one extracurricular sports activity. This narrow demographic scope restricts the diversity of the sample and overlooks potential influences of variables such as age, gender, socioeconomic status, and daily physical activity habits, which are known to impact physical fitness, cognitive function, and academic achievement. Moreover, the study did not account for the participants’ habitual physical activity levels outside structured sports, which could significantly influence fitness and cognitive outcomes. Additionally, the analysis was limited to bivariate Pearson’s correlations, without adjustments for multiple comparisons and no multivariate analyses (e.g., regression models controlling for age or BMI) were conducted. This restricts the depth of interpretation and may increase the risk of type I error. Despite these limitations, the study’s strengths include the objective measurement of VO_2_max as an indicator of physical fitness, along with comprehensive assessments of attention and concentration, thereby providing valuable insights into the complex interplay between physical health and academic performance. In this regard, it is recommended that schools consider incorporating planned exercise programs into their lessons, such as daily workouts or active learning activities. Such programs should be developed in collaboration with health experts and teachers to ensure they are suitable for all ages and abilities. Monitoring both fitness and cognitive performance would help assess the program’s effectiveness and facilitate improvements. Furthermore, focusing on a specific age group of schoolchildren would allow for more targeted insights into educational and health interventions at this developmental stage. Furthermore, focusing on a specific age group of school-aged children allows for more targeted interpretations relevant to educational and health interventions within this developmental stage. Future research should adopt longitudinal designs, incorporate larger and more diverse samples with balanced gender representation, and control for confounding variables such as habitual activity levels and socioeconomic factors, thereby offering a more robust understanding of these relationships and informing effective health promotion strategies.

## 5. Conclusions

Based on our findings, there was no overall association between physical fitness and cognitive abilities or academic performance, although there was a small positive correlation with the final PE score among male schoolchildren who participated in extracurricular sports. This makes sense since PE exerts a greater influence on physical activity and fitness, as it covers the topics and practices most closely related to these variables. However, the weak correlation observed also suggests that, while aerobic fitness (VO_2_max) contributes to PE performance, it is only one of several determinants. PE scores are likely influenced by a combination of physical, cognitive, and behavioural factors, and improvements in VO_2_max alone may not lead to substantial gains in overall PE achievement. This aligns with findings that physical fitness and academic or performance outcomes are shaped by multiple interacting variables, including lifestyle behaviours and self-esteem [3,4].

Nevertheless, it is important to note that the relationship between physical fitness and academic performance is not necessarily causal and that other factors, such as genetics, nutrition, and the environment, may also play a role. Moreover, the exact mechanisms by which physical activity and fitness influence academic performance remain unclear, necessitating further research to elucidate the relationships between these variables. Thus, future studies should be directed to explore and examine which exercise-related factors may influence cognitive abilities and academic performance. Furthermore, future intervention studies should evaluate the impact of different physical activity- and exercise-related strategies on cognitive abilities and overall academic performance. Finally, studies with a larger sample size are needed, particularly in Spain, to validate the findings of this study.

## Figures and Tables

**Table 1:** Data from anthropometric data, physical fitness, academic performance, and cognitive test scores.

Male Students (*n* = 59)				
	Mean (SD)	Range	Maximum	Minimum
Anthropometrical measures				
Age (years)	11.59 ± 1.30	5.00	15.00	10.00
Height (cm)	152.14 ± 10.49	22.00	141.00	163.00
Body mass (kg)	46.08 ± 12.26	52.00	78.00	26.00
BMI (%)	19.60 ± 3.40	15.07	28.40	13.33
Physical fitness parameters				
6MWT (m)	643.43 ± 51.99	205.00	755.00	550.00
6MWT. VO_2_max (mL/min/kg)	36.96 ± 4.65	20.59	47.34	26.75
Subjects’ score				
Total	7.19 ± 1.49	5.89	9.89	4.00
SCI	6.81 ± 1.76	7.33	10.00	2.67
LETT	6.85 ± 1.78	8.00	10.00	2.00
LAN	6.80 ± 1.85	7.00	10.00	3.00
ART	7.37 ± 1.76	7.00	10.00	3.00
PE	8.41 ± 1.23	7.00	10.00	3.00
D2 test				
TR	318.07 ± 77.58	441.00	564.00	153.00
TA	111.31 ± 60.83	499.00	511.00	12.00
O	27.00 ± 29.16	164.00	164.00	0.00
C	9.32 ± 12.74	66.00	66.00	0.00
TR+	30.69 ± 8.33	44.00	47.00	3.00
TR−	14.69 ± 6.13	27.00	27.00	0.00

Note: SCI: sciences; LETT: letters; LAN: language; ART: arts; PE: physical education; TR: processed elements; TA: successes; O: omissions; C: commissions or errors; TR+: last stimulus analysed in the row with the most attempted elements; TR−: last stimulus analysed in the row with the least attempted elements.

## Data Availability

The data presented in this study are available on request from the corresponding author due to ethical reasons.

## Abbreviations

The following abbreviations are used in this manuscript:

ART	Arts
LAN	Language
LETT	Letters
SCI	Science
PA	Physical activity
PE	Physical education
